# Efficient Recognition of Informative Measurement in the RF-Based Device-Free Localization

**DOI:** 10.3390/s19051219

**Published:** 2019-03-10

**Authors:** Jiaju Tan, Xuemei Guo, Xin Zhao, Guoli Wang

**Affiliations:** 1 Institute of Robotics and Automatic Information System, Nankai University, Tianjin 300350, China; judgetan@mail.nankai.edu.cn; 2 Tianjin Key Laboratory of Intelligent Robotics, Nankai University, Tianjin 300350, China; 3 School of Data and Computer Science, Sun Yat-sen University, Guangzhou 510006, China; guoxuem@mail.sysu.edu.cn; 4 Key Laboratory of Machine Intelligence and Advanced Computing, Ministry of Education, Sun Yat-sen University, Guangzhou 510006, China

**Keywords:** wireless sensing network, device-free localization, radio tomographic imaging, background subtraction, low-rank and sparse decomposition

## Abstract

Device-Free Localization (DFL) based on the Radio Frequency (RF) is an emerging wireless sensing technology to perceive the position information of the target. To realize the real-time DFL with lower power, Back-projection Radio Tomographic Imaging (BRTI) has been used as a lightweight method to achieve the goal. However, the multipath noise in the RF sensing network may interfere with the measurement and the BRTI reconstruction performance. To resist the multipath interference in the observed data, it is necessary to recognize the informative RF link measurements that are truly affected by the target appearance. However, the existing methods based on the RF link state analysis are limited by the complex distribution of the RF link state and the high time complexity. In this paper, to enhance the performance of RF link state analysis, the RF link state analysis is transformed into a decomposition problem of the RF link state matrix, and an efficient RF link recognition method based on the low-rank and sparse decomposition is proposed to sense the spatiotemporal variation of the RF link state and accurately figure out the target-affected RF links. From the experimental results, the RF links recognized by the proposed method effectively reflect the target-induced RSS measurement variation with less time. Besides, the proposed method by recognizing the informative measurement is helpful to improve the accuracy of BRTI and enhance the efficiency in actual DFL applications.

## 1. Introduction

Device-Free Localization (DFL) technology, which can detect the position of the target without the target carrying any electronic devices or attaching any tags, has developed rapidly in the area of assisted living [1]. As a low-cost wireless computational imaging technique to estimate the target location and protect the target privacy [2], narrowband Radio Tomographic Imaging (RTI) has been utilized in many DFL applications, such as emergency response [3], roadside surveillance [4], and assisted healthcare for the elderly [5]. As the narrowband Radio Frequency (RF) waves in RTI can penetrate through smoke, walls, and other opaque obstructions, RTI is applicable even in obstructed environments  [6]. However, as the multipath propagation is an intrinsic characteristic of the narrowband RF signal [7], the multipath interference is inevitable and seriously reduces the accuracy of RTI-based DFL [8]. How to accurately sense the target position information by effectively dealing with the negative impact of multipath interference has become a key problem in the low-cost RTI-based DFL applications [9].

Recent research has focused on how to effectively resist the multipath noise and perceive the target-induced shadow fading, containing the model-based and reconstruction-based approaches. In the model-based method, by exploring the characteristic of RSS variation by target-induced attenuation, some fine-grained projection models have been analyzed to evaluate the impact of target-induced shadow fading [10,11,12]. Though the fine-grained RSS change of target-induced shadow fading can be described by the model-based method, the projection model should be re-estimated in different experimental scenes so that the practicability is limited. In the reconstruction-based method, as the target only occupies a small area in the sensing network, the target-induced shadow fading can be treated as sparse [13]. Then, the methods based on sparse reconstruction, such as Compressive Sensing [14], Sparse Bayesian Leaning [15], and spatiotemporal Sparse Bayesian Leaning [16], are applied for RTI reconstruction. Meanwhile, the calculation for reconstructing the high-dimensional image is so high that the real-time DFL performance is limited. To overcome this time-consuming problem of solving the under-determined equations in RTI directly, the Back-projection RTI (BRTI) has been proposed to realize the lightweight imaging for DFL applications [17,18]. It is verified that the shadow fading can be regarded as the weighted sum of RF link attenuation. When these truly target-affected RF links with their influence can be found fast and precisely, the target-induced shadow fading can be effectively reconstructed just by the linear combination of the effective RF links. Therefore, by transforming the issue of reconstructing the high-dimensional shadow fading into a problem of low-dimensional RF link selection, BRTI only deals with the RSS measurement of the fixed low dimension rather than the shadow fading in the high dimension. Hence, BRTI may obtain higher accuracy when the monitored area is divided into more fine-grained grids without increasing the time complexity. However, the contributive RF link measurements found have a certain number and depend on precise projection models. Besides, the number of effective RF links to sense in the target-induced dynamic RSS change is uncertain. Further, the precise projection models should be pre-estimated and are unpractical in actual DFL applications. Therefore, a method that can accurately perceive the time-varying RSS variation and efficiently recognize the truly target-affected RF links is helpful to resist the multipath interference and improve the performance of BRTI reconstruction.

Numerous studies have been proposed to figure out the attenuated RF links affected by target appearance. To evaluate the attenuation of the RF link, the variance of RSS is a simple measure, but the accuracy is coarse, as it is sensitive to the multipath noise [6]. The RF link measurement uncertainty can be a reasonable measure but lacks efficiency due to the complex computation [19]. To quantify effectively the target-induced effect, background subtraction from computer vision [20] has been used for the contributive RF link recognition [21], in which the target-affected RF links with a large change of RSS are regarded as the foreground links and the rest of the RF links are treated as the background links [22]. Then, the RSS distribution is used to estimate the different RF link states, but the distribution estimation is sensitive to the multipath interference and leads to RF link misjudgment [22,23]. The link subtraction based on the spatial similarity [21] can cope with some misjudged RF links but consumes much time for calculation. Thus, a method that accurately perceives the fine-grained RSS variation by target appearance and efficiently realizes the RF link state decomposition is helpful for the informative measurement recognition is still lacking according to the literature.

To analyze the spatiotemporal variation of RF links in real-time tracking, this work treats this RF link state decomposition as a matrix decomposition problem by putting the measured RSS in the tracking process into a matrix. Then, based on the background subtraction [21], this RSS matrix can be decomposed into the background and foreground parts. Considering the process of target movement, in view of the temporal continuity and the limited spatial range, only a few RF links that are closest to the target may change greatly so that the target-induced foreground links are spatially sparse. On the contrary, the remaining RF links may remain almost unchanged, so that the columns of the background matrix are approximately equal to each other, which indicates that the background matrix can be treated as temporally low-rank. Therefore, this article proposes a method based on the low-rank and sparse decomposition [24] to sense precisely and quickly the spatiotemporal variation of RSS using Principal Component Pursuit (PCP) [25]. By the proposed method, the informative RF links can be effectively recognized and used to enhance the reconstructed performance of BRTI. Furthermore, both the accuracy and efficiency in real-time DFL and tracking application can be also improved.

The paper is organized as follows. Section 2 is a brief description of the RTI reconstruction issue, the reconstruction principle of BRTI, and the RF link selection based on background subtraction. In Section 3, the proposed method based on the low-rank and sparse decomposition is given to realize the informative RF link selection and BRTI reconstruction. In Section 4, the experimental design including the indoor and outdoor tracking is deployed. In Section 5, the experimental results, including the recognized informative RF links, the reconstruction of shadow fading, and the RF link analysis, are given to show the effectiveness and efficiency of the proposed method to perceive the informative measurements and improve the BRTI reconstruction. The conclusion of this paper is in Section 6.

## 2. Related Work

### 2.1. Radio Tomographic Imaging

**Figure 1:**
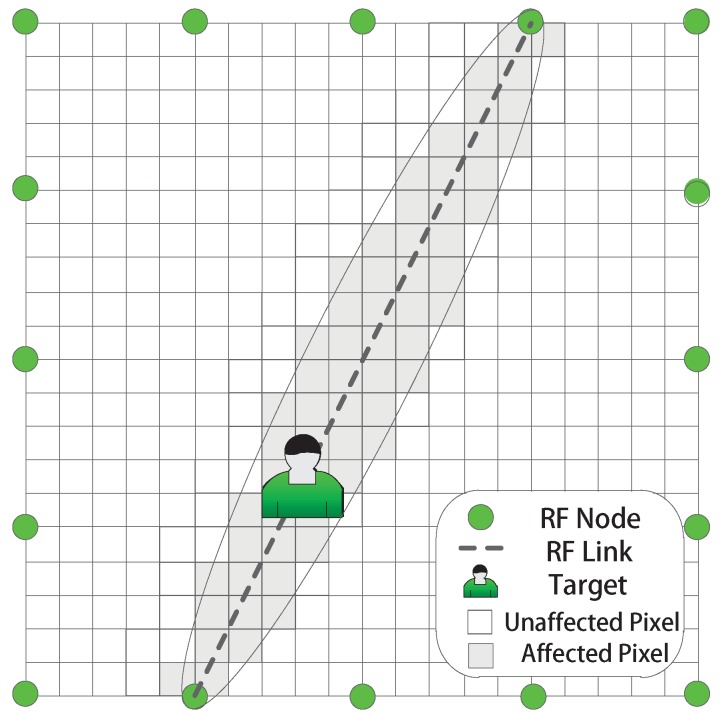
An illustration of the RF network in RTI.

A brief description of RTI and an illustration of RTI is shown in Figure 1. In the monitored area, an RF sensing network covered by the signal transition between the RF nodes is deployed. When the target comes into the network, some RF links in the area may be interfered or faded, and this fading results in the change of the Received Signal Strength (RSS). Hence, RTI reconstructs the object-induced shadow fading from the measured RSS. Then, the target position information, which is implicit in the reconstructed image from shadow fading, can be extracted. In general, considering a 2D monitored area in which *m* sensors are deployed along the perimeter, then an equipotent sensing network containing M=m(m−1)/2 RF links is constructed. The monitored area can be divided into *N* virtual pixels, then the vector $x\in R^{N}$ represents the target-induced shadow fading in the sensing network. Then, the RSS variation of each RF link, which forms the measurement vector $y\in R^{M}$, is the quantitative measurement to reflect the shadow fading. The RSS can be estimated as the weighted sum of shadow fading through the projection equation as:

(1) $$y_{i}=\sum_{j=1}^{N}\Phi_{i,j}x_{j}+e_{i},$$

where $e\in R^{M}$ is the measurement noise. $\Phi ={\left[\phi_{1},\ldots ,\phi_{M}\right]}^{T}$ is the projection matrix, as the quantitative relationship between RSS variation and shadow fading. $\Phi_{i,j}$ represents the projection impact of shadow fading at pixel *j* to the RSS of link *i* as:

(2) $$\Phi_{i,j}=\frac{1}{\sqrt{d_{i}}}\left\{\begin{array}{cc} 1,\; & \mathrm{if}\;d_{ij}^{\mathrm{tran}}+d_{ij}^{\mathrm{rec}}<d_{i}+\gamma ,\\ 0,\; & \mathrm{otherwise}, \end{array}\right.$$

where $d_{i}$ is the length of the link *i* and $d_{ij}^{\mathrm{tran}}$ and $d_{ij}^{\mathrm{rec}}$ are the distance from the centroid of pixel *j* to the transmitter and receiver of link *i*. The parameter γ controls the range of the target-affected projection zone and only the pixels in the projection zone are considered as affected [2,26], as Figure 1 shows. The shadow fading induced by the target appearance will lead to the RSS variation of the RF link. Then, the prime goal of RTI is to reconstruct the image of shadow fading x and infer the position of the target by solving the projection Equation (1) from the RSS y. Due to the narrow-band property of the RF signal, the multipath noise in the sensing network increases the uncertainty of the measured RSS [8]. These interfered measurements induced by the multipath fading may degrade the reconstruction quality. Therefore, the multipath interference is a key problem in actual RF-based DFL applications.

### 2.2. Back-Projection Radio Tomographic Imaging

The RSS measurement (1) of link *i* can be formulated as:

(3) $$y_{i}=\sum_{j=1}^{N}\Phi_{i,j}x_{j}+e_{i}={\phi_{i}}^{⊤}x+e_{i},$$

so that this reconstruction issue of x from the measurement Equation (1) can be treated as a regression problem, where ${\left\{\left({\phi_{i}}^{⊤},y_{i}\right)\right\}}_{i=1}^{M}$ can be regarded as the training data. Then, by transforming this regression problem into a support vector regression optimization [27], the optimal x can be acquired as:

(4) $$x=\sum_{i=1}^{M}z_{i}\phi_{i}=\Phi^{⊤}z,$$

where $z={\left[z_{1},\cdots ,z_{M}\right]}^{⊤}$ is the weight that should be determined in BRTI [17].

Equation (4) shows that the shadow fading x is the linear combination of RF links with the weight z. The $z_{i}$ with large absolute value shows that this RF link *i* contributes greatly to the fading image x. Meanwhile, the $z_{i}$ with a small absolute value implies that this RF link *i* has little effect on the fading image x. Then, z can be regarded as the contribution of RF links to integrate the shadow fading x. Besides, as only a minority of RF links are affected by the target appearance, the z is also sparse. In comparison, Equation (1) measures the effect on RF links of the fading image, while Equation (4) evaluates the reaction to the fading image from RF links. Thus, Equation (4) can be named back-projection imaging because it can obtain the shadow fading x from the contribution z of RF links.

BRTI is a useful method to achieve efficient location estimation. To estimate the target-induced shadow fading x, direct imaging is used for the underdetermined Equation (1). The resolution and accuracy of RTI can be improved by increasing the pixels in the sensing network. However, the dimension of x will become so large that solving Equation (1) would be inefficient for real-time applications due to its high time complexity. Comparatively, BRTI directly analyzes the measured data y to figure out the contributive RF links with their contribution z to estimate the shadow fading x by just a simple linear transformation. As the dimensions of y and z are low and fixed, the complexity of back-projection imaging remains unchanged as the dimension of x increases. When the informative RF links can be figured out, the back-projection imaging is helpful for the efficient and accurate DFL application. Thus, the key problem of BRTI is to find out the RF links with the contribution z from the measured RSS data y. Further, the sparsity of z indicates that the least, but essential RF links with their contribution should be figured out to realize the fast BRTI. To achieve this goal, a method that can efficiently and accurately perceive the informative RF links is useful for BRTI and DFL applications.

### 2.3. RF-Link Recognition Based on Background Subtraction

**Figure 2:**
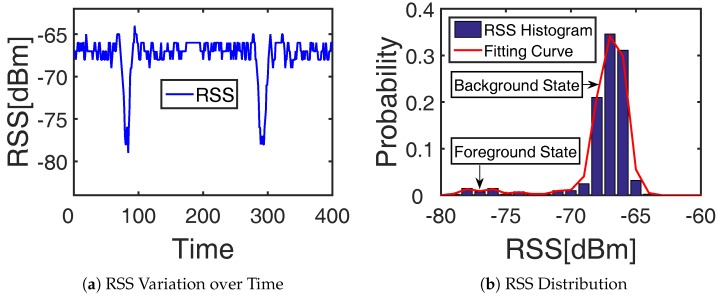
Characteristic variation of a chosen RF link.

To figure out the informative RF links, it is important to analyze whether the RF link is affected by the target. Here, we give an analysis of an RF link that is crossed by a target twice. The RSS variation of this chosen link over time is shown in Figure 2a. The RSS varies a little within the difference of 3 dBm for most of the time when the target is far away from this RF link while the RSS decreases more than 15 dBm when the target comes close to this RF link. The histogram and fitting curve of the RSS distribution in Figure 2b show that the RF link will be in different states depending on whether it is target-affected or not. Thus, the issue of target-induced RF link recognition is actually the analytical problem of the RF link state. Considering the dynamic variation of the RF link state, background subtraction from computer vision [20] can be applied to this RF link state analysis [21]. The RF link state with high peak and low variance is treated as the static background state, and the other RF link state with low peak and large variance is regarded as the dynamic foreground state [22], as Figure 2b shows. Then, the target-induced RF link recognition can be realized by the background and foreground decomposition of the RF link state.

Various methods based on component analysis are utilized to handle the decomposition problem of the RF link state. The motion-induced variance is used to simply reflect the foreground link state [6], and the kernel distance is applied to estimate the RSS distribution directly [26]. Considering the dynamic variation of the RSS distribution, Kernel Density Estimation (KDE) [23] and the Mixture of Gaussians (MoG) [22] can improve the link-state learning by estimating the background-link probability. Multipath interference exists in the actual environment, so the distribution of RSS may be too complex and time-consuming to be fitted by an appropriate model. To improve the results of MoG and KDE, which only estimate the temporal variation of RSS, based on the spatial similarity between the adjacent links [21], Link Subtraction (LS) analyzes the RF link state and additively subtracts the misjudged target-induced links, which are actually far away from the target. However, LS consumes much time for calculation and merely considers the spatial variation of the RF link state so that it is incapable of dealing with the multipath links, which continue to exist during the measurement process of target movement. Thus, a method that can accurately and quickly recognize the RF link state by simultaneously perceiving the spatiotemporal RSS variation in actual DFL application is needed.

## 3. Proposed Method

In the real-time tracking process, to recognize the spatiotemporal variation of the RF links effectively, the total measured RSS of all the RF nodes during the measuring time can be recorded in a matrix to reflect the spatiotemporal change of RSS. The RSS of the RF links during the measuring process can be recorded in a matrix as $Y=\left[y_{1},\cdots ,y_{T}\right]\in R^{M\times T}$, with the element $Y_{i,t}$, meaning the RSS of the link *i* at the sampling time *t*. The row number *M* is the total number of links, and the column number *T* is the total sampling time. The vector $y_{t}\in R^{M}$ can be transformed into an $R^{M\times M}$ matrix to express the spatial variation of the RF link state at different RF nodes. The difference $y_{t}$ shows the temporal variation of the RF link state at each measuring time. The state analysis issue of the RF links is to decompose the measurement matrix *Y* into the background-link matrix and foreground-link matrix as:

(5) Y=L+S,

where *L* is the stationary background-link component and *S* is the time-varying foreground portion.

While only a few links are target-affected to become the foreground links, then $S_{:,t}$, the RSS of foreground links at time *t*, includes only a small amount of large values. On the contrary, $L_{:,t}$, the RSS of background links at time *t*, changes little between the adjacent time so that $L_{:,t+1}$ is approximate, so as to be the linear representation of $L_{:,t}$. Then, *S* and *L* in (5) can be regarded as sparse and low-rank, so (5) can be transformed into a low-rank and sparse decomposition problem of matrix *Y*, as:

(6) $$\min_{L,S}\;\mathrm{rank}\left(L\right)+{∥S∥}_{0}\;s.t.\;Y=L+S,$$

which can be solved based on Principal Component Pursuit (PCP) [24,28].

The $\ell^{0}$ norm minimization problem of *S* in (6) is non-convex and NP-hard, as the $\ell^{1}$ norm is the convex surrogate of the $\ell^{0}$ norm, so it can be relaxed as an $\ell^{1}$ norm minimization problem. Then, (6) can be transformed into an optimization problem [28] under a low-rank constraint as:

(7) $$\min_{L,S}\;\frac{1}{2}{∥L+S-Y∥}_{F}^{2}+\lambda {∥S∥}_{1}\;s.t.\;\mathrm{rank}\left(L\right)\leq r,$$

where λ is the regularization parameter and ${∥\cdot ∥}_{F}$ and ${∥\cdot ∥}_{1}$ are the Frobenius norm and the $\ell^{1}$ norm.

*L* and *S* in (7) can be solved iteratively by two sub-problems. At first, *L* is estimated by solving this rank constraint optimization problem as:

(8) $$L^{\left[k+1\right]}=\underset{L}{\arg\min}{∥L+S^{\left[k\right]}-Y∥}_{F}\;s.t.\;\mathrm{rank}\left(L\right)=r.$$

where [k] means the $k^{\mathrm{th}}$ iteration. It can be solved by a partial SVD of $\left(Y-S^{\left[k\right]},r\right)$ with *r* components with *r* from 1–4 [25] and rank-one modification [29] to control the rank, as:

(9) $$\begin{array}{cc} \left[U^{\left[k+1\right]},\Sigma^{\left[k+1\right]},V^{\left[k+1\right]}\right] & =\mathrm{SVD}(Y-S^{\left[k\right]}),\\ L^{\left[k+1\right]} & =U_{:,1:r}^{\left[k+1\right]}*\Sigma_{1:r,1:r}^{\left[k+1\right]}*V_{:,1:r}^{[k+1]⊤}. \end{array}$$

Then, *S* is obtained by solving the $\ell^{1}$ minimization problem:

(10) $$S^{\left[k+1\right]}=\underset{S}{\arg\min}\frac{1}{2}{∥L^{\left[k+1\right]}+S-Y∥}_{F}+\lambda {∥S∥}_{1},$$

which can be solved by the soft thresholding operation: [30],

(11) $$S^{\left[k+1\right]}=\mathrm{sign}\left(Y-L^{\left[k+1\right]}\right)\max(0,∥Y-L^{\left[k+1\right]}-\lambda {∥}_{F}),$$

where sign is the signum function, defined as:

(12) $$\mathrm{sign}\left(\delta \right)=\left\{\begin{array}{cc} 1 & ,\delta >0,\\ 0 & ,\delta =0,\\ -1 & ,\delta <0. \end{array}\right.$$

**Algorithm 1:** The recognition of informative RF links and use for BRTI reconstruction based on PCP. **Control Variables:** RSS Matrix $Y\in R^{M\times T}$           Regularization Parameter λ=0.0375,           Eigenvalue Threshold $\tau =10^{-6}$,           Link State Threshold θ=0.6,            Iteration Restriction K=3,           Sampling Windows $T_{0}=25$, **Initialization:** Initial Rank of *L*, r=1,         Background Link Matrix $L=Y_{:,1:T_{0}}$,         Foreground Link Matrix $S=0^{M\times T_{0}}$,   1: **for**
t=26;t<T;t++
**do**  2:  **for**
k=1;k<K;k++
**do**  3:   $\left[U^{\left[k\right]},\Sigma^{\left[k\right]},V^{\left[k\right]}\right]=\mathrm{SVD}\left(Y_{:,t-T_{0}+1:t}-S^{\left[k\right]}\right)$;  4:   $L^{\left[k\right]}=U_{:,1:r}^{\left[k\right]}*\Sigma_{1:r,1:r}^{\left[k\right]}*V_{:,1:r}^{[k]⊤}$;  5:   **for**
l=1;l<r;l++
**do**  6:    $v_{l}=\Sigma_{l,l}^{\left[k\right]}$;  7:   **end for**
  8:   **if**
$\frac{v_{r}}{\sum_{l=1}^{r}v_{l}}>\tau ,\;r<4$
**then**  9:    r=r+1;  10:   **end if**  11:   $S_{1}^{\left[k+1\right]}=\mathrm{sign}\left(Y_{:,t-T_{0}+1:t}-L^{\left[k+1\right]}\right)$;  12:   $S_{2}^{\left[k+1\right]}=\max(0,∥Y_{:,t-T_{0}+1:t}-L^{\left[k+1\right]}-\lambda {∥}_{F})$;  13:   $S^{\left[k+1\right]}=S_{1}^{\left[k+1\right]}S_{2}^{\left[k+1\right]}$;  14:  **end for**  15:  $L_{:,t}=L_{:,T_{0}}^{\left[k\right]}$;  16:  $S_{:,t}=S_{:,T_{0}}^{\left[k\right]}$;  17:  **for**
i=1;i<M;i++
**do**  18:   $p\left(B_{i,t}|Y_{i,t}\right)=\frac{L_{i,t}-\min\left(L_{:,t}\right)}{\max\left(L_{:,t}\right)-\min\left(L_{:,t}\right)}$;  19:   **if**
$p(B_{i,t}|Y_{i,t})<\theta$
**then**  20:    i∈F;  21:   **else**  22:    i∈B;  23:    $S_{i,t}=0$;  24:   **end if**  25:  **end for**  26:  $X_{:,t}=\Phi^{⊤}S_{:,t},i\in F$;  27: **end for**

After the background link matrix *L* is obtained, a link *i* at time *t*, the posterior probability of being in the background-link state can be calculated as:

(13) $$p\left(B_{i,t}|Y_{i,t}\right)=\frac{L_{i,t}-\min\left(L_{:,t}\right)}{\max\left(L_{:,t}\right)-\min\left(L_{:,t}\right)}.$$

A criterion threshold θ=0.6 is set to determine whether the link *i* belongs to the foreground link set F or the background link set B. i∈F when $p(B_{i,t}|Y_{i,t})<\theta$ and l∈B when $p(B_{i,t}|Y_{i,t})⩾\theta$ otherwise. Then, the link state decomposition is completed, and i∈F are the informative RF links. Finally, the image of shadow fading can be reconstructed by BRTI using the recognized informative RF links as:

(14) $$X_{:,t}=\Phi^{⊤}S_{:,t},$$

where only the element $S_{i,t}$ with i∈F in $S_{:,t}$ is used for BRTI reconstruction. The whole PCP method for the recognition of informative RF links and the BRTI reconstruction is shown in Algorithm 1.

## 4. Experiment Design

**Figure 3:**
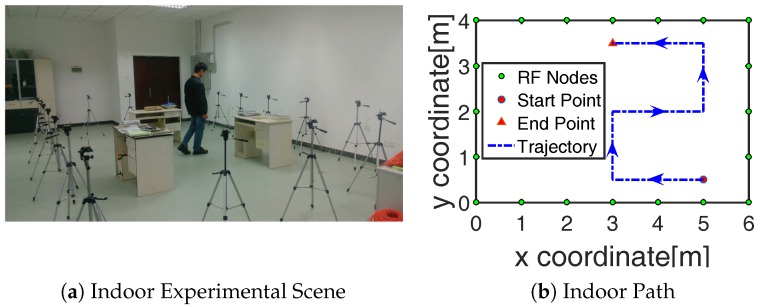

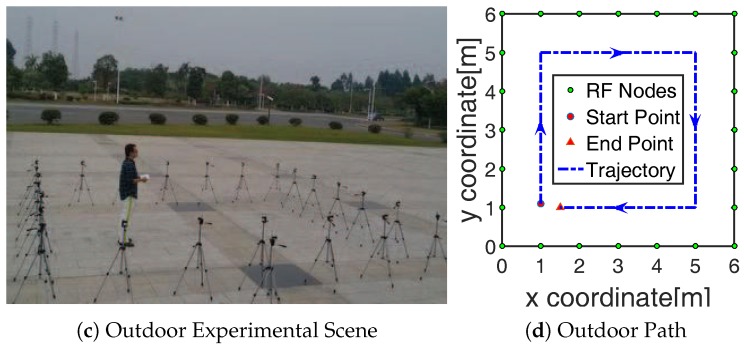
Experiment deployment.

The experiments were deployed in a 4 m × 4 m indoor scene with 20 RF nodes and a 6m×6 m outdoor scene with 24 RF nodes in Sun Yat-sen University, shown in Figure 3a,c The RF nodes along the boundary at a height of 1 m with an interval of 1 m were the Crossbow MICAz devices, which communicate with a 2.4-GHz IEEE 802.15.4 standard and are suitable for RTI research [2,7]. A token ring protocol was used so that each RF node transmitted the RF signal in sequence according to the predefined ID number. When an RF node was transmitting the signal, the rest of the RF nodes remained in receiver mode and recorded the RSS measurements from the received packets. A MIB520CB base station made by Crossbow overheard all the packets and passed them to a computer for storage. The period for token transmission was 120 ms. Before the localization experiment, the baseline RSS value $y_{0}$, which reflects the RSS in the empty monitored area, was calculated as the average RSS measurements of 5 min before the target entered the scene. During the localization experiment of each position, the RSS averaged over a 30-s period was used as the measured RSS value $y_{1}$. Then, the RSS for RF link state analysis and BRTI reconstruction was $y=y_{1}-y_{0}$. The parameter γ=0.04, which is reasonable to reflect the target-affected projection area as discussed in [11]. The size of a pixel in the sensing area was 0.1 m×0.1 m, and the total pixel number in the indoor and outdoor scene was N=2400 and N=3600, respectively. The number of RF links in the indoor and outdoor scene was M=190 and M=276, respectively. Then, a target moved with a uniform motion along the prescribed destination path inside the network, as the blue trajectory line shown in Figure 3b,d. The total number of sampled points for tracking in the indoor and outdoor scene was T=250 and T=400, respectively.

## 5. Experiment Results

The performance of the proposed method was compared with KDE-LS and MoG-LS, which are the combinational methods by using the Link Subtraction (LS) modification [21] to improve the estimated results by KDE [23] and MoG [22]. The experimental results for comparison included the recognition results of the target-affected foreground-state RF Links (Section 5.1), the localization results based on BRTI reconstruction by the recognized RF links (Section 5.2), and the sensitivity analysis of an RF link (Section 5.3).

### 5.1. Recognized Results of Foreground-State RF Links

**Figure 4:**
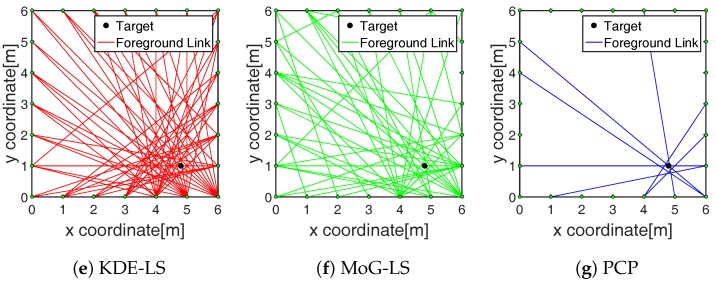
Recognized foreground links of different methods.

The number of foreground-state RF links estimated by these methods are recorded in the third row of Table 1. Following is an example to show the recognition results of foreground links. A target, which moved to the point (4.9,1.0) in the outdoor scene Figure 3d, was analyzed. The foreground-state RF links recognized by the KDE-LS, MoG-LS, and PCP are shown in Figure 4a–c, respectively. The foreground-state RF links recognized by PCP were tightly located around the target. Meanwhile, the RF links recognized by KDE-LS and MoG-LS included many redundant RF links far from the target. As the RF links that were close to the target would be greatly faded by the target appearance, the least but most informative foreground links were perceived by PCP. This comparison of the recognized foreground links indicates that the proposed PCP method based on the low-rank and sparse decomposition can precisely sense the truly target-affected RF links without inducing redundant links far from the target.

### 5.2. Reconstructed Image Based on the Informative RF Links

**Figure 5:**
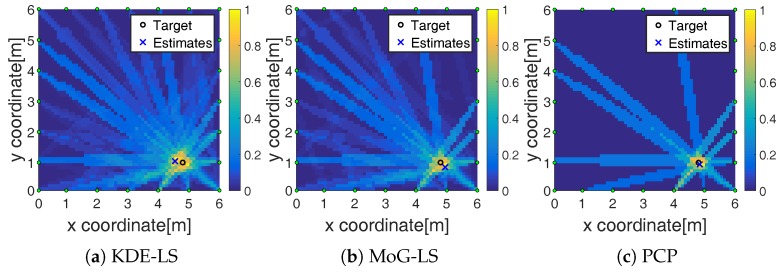
Reconstructed fading image by BRTI of different methods.

After the foreground-state RF links have been recognized, the image of shadow fading can be reconstructed by BRTI from the obtained foreground-state RF links. In the above example, when the target moves to the point (4.9,1.0) in the outdoor scene, Figure 3d, the images of shadow fading reconstructed based on the recognized RF links are shown in Figure 5. From these results, the fewest foreground-state RF links estimated by the proposed PCP method were sufficient to reconstruct the image of shadow fading without bringing in the noise-induced artifact, meaning that the RF links recognized by PCP effectively reflected the target-induced shadow fading. Besides, the target position was computed as the coordinate with the maximum in the estimated image of shadow fading ${\hat{x}}_{t}={\left[x_{1,t},\cdots ,x_{N,t}\right]}^{T}$ as:

(15) $$k_{t}={\arg\max}_{j}x_{j,t},$$

then ${\hat{w}}_{t}=\left(p_{k_{t}},q_{k_{t}}\right)$ in the centroid coordinate of pixel *k* is the estimated location. The localization error is the root mean squared error between all the estimated positions $\hat{w}={\left[{\hat{w}}_{26},\cdots ,{\hat{w}}_{T}\right]}^{T}$ and the true positions $w={\left[w_{26},\cdots ,w_{T}\right]}^{T}$ as:

(16) $$d_{t}=\sqrt{\frac{1}{T-25}\sum_{t=26}^{T}{\left({\hat{w}}_{t},w_{t}\right)}^{2}}.$$

**Figure 6:**
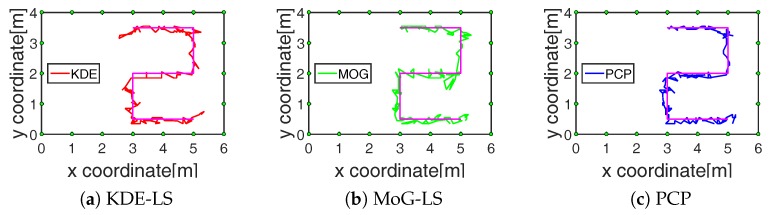
Indoor tracking results of different methods.

**Figure 7:**
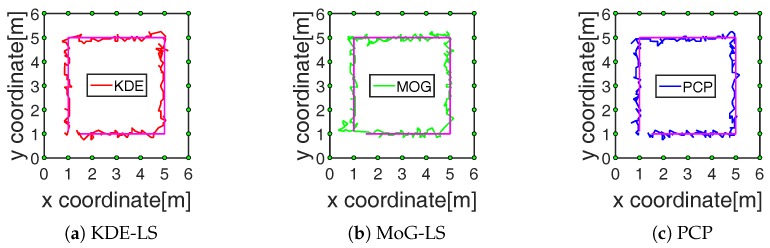
Outdoor tracking results of different methods.

The performance of these methods in the tracking process, including 250 and 400 points in the indoor and outdoor scene, respectively, were compared with the tracking results presented in Figure 6 and Figure 7. The average calculating time and the average tracking error in the total tracking process by these methods are recorded in the fourth and fifth row of Table 1. On the one hand, the time consumption of PCP was decreased by at least 33% and 53% compared to KDE-LS and MoG-LS. On the other hand, the localization accuracy of PCP was increased by at least 2.6 cm and 1.7 cm compared to the other methods. These results demonstrate that the recognition of the foreground RF links by PCP was effective and efficient to reflect the target-induced shadow fading. Meanwhile, KDE-LS and MoG-LS, based on the temporal distribution estimation and the spatial link subtraction led to the higher error and more computation than PCP. This is because the time window of the distribution estimation in KDE-LS or MoG-LS cannot be adaptively changed to sense the time-varying scene, and the spatial link subtraction for the overall calculation was not only time-consuming, but also ineffective for the RF links that still existed in the sensing area. In contrast, the low-rank and sparse decomposition of the measured RSS matrix by PCP can precisely sense the spatiotemporal variation of shadow fading simultaneously. Thus, more accurate position information of shadow fading can be found by the proposed method.

### 5.3. RF Link Analysis

**Figure 8:**
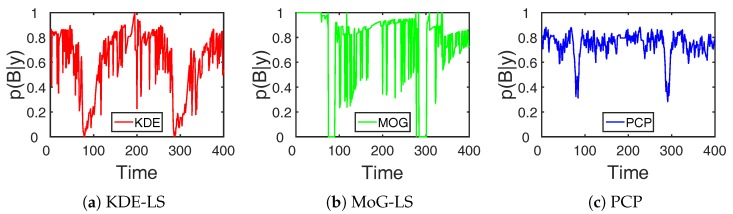
Background-link state probability of different methods.

Moreover, the sensitivity analysis of the RF link state was conducted to demonstrate the fine-grained perception ability of these methods. The background-link state probability (13) in the RF link state decomposition was used to approximate the real-time RSS variation of the RF link. To analyze the RF link RSS variation in Figure 2a, the background-link state probability estimated by these methods is shown in Figure 8. The Pearson correlation coefficient is a measure of the similarity between the background-link state probability and the RSS change, with the results recorded in the fourth row of Table 1. From the graph and correlation results, the background-link state probability estimated by PCP was the closest to the RSS change with the highest Pearson correlation coefficient. These results indicate that the proposed PCP method accurately recognized the large RSS change by the target appearance and was insensitive to the small RSS change caused by the environmental noise. Thus, this RF link recognition by the low-rank and sparse decomposition was informative and robust to the multipath interference.

### 5.4. Discussion

The RF link recognition results by our proposed method based on PCP were not only closest to the target with the lowest count, but also the fastest compared to the other methods as recorded in the third and fourth row of Table 1 and shown in Figure 4. Besides, the localization error by BRTI reconstruction based on the recognized RF links by the proposed method was also the least in the comparison, as recorded in the fifth row of Table 1 and shown in Figure 5, Figure 6 and Figure 7. The improved accuracy and efficiency were obtained because the spatiotemporal variation of RF links can be sensed simultaneously by the proposed method, as the RF link sensitivity analysis recorded in the sixth row of Table 1 and shown in Figure 8. Meanwhile, the temporal distribution estimation and spatial similarity analysis of RF link by KDE-LS and MoG-LS were sensitive to interference and time-consuming. Moreover, as the resolution of BRTI and the time complexity of the informative RF link recognition was based on the size of the area of interest and the total number of RF links, the error and the time consumption in the small indoor scene were both smaller than those in the big outdoor scene. Furthermore, as there were more complex multipath components in the indoor scene than the outdoor scene [7,9], the recognized RF links being affected in the indoor scene were more than those in the outdoor scene, which will be analyzed in our future research.

## 6. Conclusions

The RF link mismeasurements induced by the multipath noise often degrades the reconstructed RTI quality and decreases the DFL accuracy. Thus, the recognition of the crucial RF link measurements that are truly affected by the target appearance is effective to resist the multipath interference. For better recognition of the contributive RF links, this paper proposes a method based on the low-rank and sparse decomposition to perceive the spatiotemporal variation of the RF link state rather than coping with the temporal and spatial RSS variation separately in the commonly-used methods. From the experimental results, the least but most informative RF links figured out by the proposed method are more accurate and efficient to reflect the target-induced shadow fading and improve the quality of BRTI reconstruction. Besides, the proposed method shows its effectiveness for the fine-grained dynamic analysis of RF signals and contributes to the RF-based real-time DFL applications.

## Figures and Tables

**Table 1:** Quantitative analysis of different methods.

Scene	Indoor			Outdoor		
Method	KDE-LS	MoG-LS	PCP	KDE-LS	MoG-LS	PCP
Links	97	76	**12**	85	68	**9**
Time(s)	0.560	0.801	**0.383**	0.623	1.309	**0.405**
Error(m)	0.257	0.248	**0.231**	0.291	0.285	**0.264**
Correlation	0.547	0.634	**0.743**	0.685	0.714	**0.851**

## Abbreviations

The following abbreviations are used in this manuscript:

BRTI	Back-projection Radio Tomographic Imaging
RF	Radio Frequency
DFL	Device-Free Localization
RSS	Received Signal Strength
KDE	Kernel Density Estimation
MoG	Mixture of Gaussians
LS	Link Subtraction
PCP	Principal Component Pursuit
